# Engineering Immortalized Bovine Granulosa Cells Using a Triple‐Gene Approach: Mutant CDK4, Cyclin D1, and TERT

**DOI:** 10.1002/cbin.70126

**Published:** 2026-01-21

**Authors:** Lanlan Bai, Minami Takahashi, Jin Kobayashi, Takahiro Eitsuka, Himari Matsusaka, Taku Ozaki, Eriko Sugano, Hiroshi Tomita, Yuan Xu, Kazuhiro Kawamura, Kiyotaka Nakagawa, Tohru Kiyono, Tomokazu Fukuda

**Affiliations:** ^1^ Department of Life Sciences, Faculty of Agriculture Iwate University Morioka Iwate Japan; ^2^ Graduate School of Science and Engineering Iwate University Morioka Iwate Japan; ^3^ School of Food Industrial Sciences Miyagi University Sendai Miyagi Japan; ^4^ Graduate School of Agricultural Science Tohoku University Aoba‐ku Sendai Japan; ^5^ Department of Obstetrics and Gynaecology Juntendo University Faculty of Medicine Bunkyo‐ku Tokyo Japan; ^6^ Exploratory Oncology Research & Clinical Trial Center National Cancer Center kashiwanoha Chiba Japan; ^7^ Department of Cancer Cell Research Sasaki Institute Chiyoda‐ku Tokyo Japan

**Keywords:** aromatase, cattle, granulosa cells, immortalization, K4DT

## Abstract

Advancing reproductive technologies in livestock is essential to improve both productivity and genetic potential of cattle. Despite this importance, application of reproductive biotechnologies in cattle breeding remains limited. Bovine granulosa cells (bGCs), which are key components of the ovarian follicle, are critical in female reproduction as they produce steroid hormones and growth factors necessary for oocyte development. However, primary bGCs exhibit restricted proliferative capacity in vitro, limiting their utility in large‐scale studies on mechanisms related to follicular development. To address this limitation, we attempted to immortalize bGCs by co‐expressing human mutant cyclin‐dependent kinase 4 (CDK4^R24C^), cyclin D1, and telomerase reverse transcriptase (TERT) using lentiviral vectors. The resulting immortalized cells (bGCs‐K4DT) displayed extended proliferative lifespans, surpassing 100 population doublings without exhibiting signs of senescence. The transduced cells demonstrated a more active cell cycle profile and higher telomerase activity relative to parental bGCs. Importantly, they retained the bGC‐specific marker, aromatase, albeit at reduced expression levels. This immortalized bGC offers a promising model for investigating the role of bioactive components of platelet‐rich plasma (PRP) in follicular activation and growth, thereby supporting innovations in livestock reproductive technologies.

**Abbreviations**
bGC: bovine granulosa cells
CDK4R24C: mutant cyclin‐dependent kinase 4
CYP19A1: cytochrome P450 family 19 subfamily A member 1
E2: estrogen
EGFP: enhanced green fluorescent protein
FSH: follicle‐stimulating hormone
K4D: mutant cyclin‐dependent kinase 4 with cyclin D1
K4DT: mutant cyclin‐dependent kinase 4 with cyclin D1 and telomerase reverse transcriptase
LH: luteinizing hormone
P450scc: cholesterol side‐chain cleavage enzyme
PD: population doubling
pRB: retinoblastoma protein
PRP: platelet‐rich plasma
qRT‐PCR: quantitative real‐time PCR
SA‐β‐Gal: senescence‐associated β‐galactosidase
SV40: Simian Virus 40
TERT: telomerase reverse transcriptase
TSC1: tuberous sclerosis complex 1
yGC‐SV40T: yak granulosa line
∆∆CT: comparative CT

## 1 Introduction

Granulosa cells are somatic cells that are located within the ovarian follicles of female mammals. They envelop the developing oocytes and provide structural support and metabolic nourishment throughout follicular maturation (McGee and Hsueh 2000). Under the influence of follicle‐stimulating hormone (FSH), granulosa cells express the enzyme aromatase, which enables conversion of androgens (supplied by theca cells) into estrogens (Simpson and Davis 2001). Following ovulation, these cells undergo luteinization and transform into luteal cells that synthesize and secrete progesterone, which is essential for maintaining the endometrial lining and supporting early pregnancy (Robker and Richards 1998). In the context of livestock management, advancing reproductive technologies is critical for boosting productivity and enhancing the genetic potential of cattle. A promising innovation in this field is the application of platelet‐rich plasma (PRP), which has shown a potential to stimulate ovarian follicle activation, thereby increasing both the quantity and quality of retrieved oocytes (Cremonesi et al. 2020). Despite the promising outcomes associated with the application of PRP, there remains a lack of robust in vitro systems to evaluate its efficacy and elucidate the underlying mechanisms of action. Bovine granulosa cells (bGCs), which are key regulators of follicular development and hormone production, are integral to this field of research (Lange‐Consiglio et al. 2023). Developing an immortalized bGC provides a valuable model to investigate ovarian physiology and test reproductive technologies, such as PRP therapy (Seckin et al. 2022). Unlike primary cell cultures, immortalized cells bypass senescence, enabling consistent and scalable experimentation (Lerner et al. 1995).

Recently, the K4DT method, which utilizes simultaneous expression of human mutant cyclin‐dependent kinase 4 (CDK4^R24C^), cyclin D1, and telomerase reverse transcriptase (TERT), has been proven to be effective in conferring limitless proliferative capacity to a range of mammalian cell types (Bai et al. 2023; Bai et al. 2024; Fukuda et al. 2021; Kikuchi et al. 2023; Furuya et al. 2021). The CDK4–cyclin D1 complex functions as a kinase that phosphorylates and inactivates the retinoblastoma protein (pRB). Under conditions of stress induced by cell culture, the CDK inhibitor, p16, is upregulated and binds to the CDK4–cyclin D1 complex, thereby inhibiting its activity and triggering premature senescence. The mutant form of CDK4, CDK4^R24C^, retains the ability to associate with cyclin D1 but is resistant to binding to the protein, p16. Consequently, the CDK4^R24C^–cyclin D1 complex remains active, phosphorylates pRB, and circumvents the onset of premature senescence (Rane et al. 2002). Traditional immortalization methods, such as transducing cells with Simian Virus 40 (SV40) large T antigen, function by inactivating pRB and p53 (the latter is known as a guardian of the genome); this process allows cells to tolerate DNA damage caused by oncogene activation (Lin and Simmons 1991; Lundberg et al. 2000). However, the expression of these oncogenes often induces chromosomal abnormalities, resulting in SV40‐immortalized cells exhibiting characteristics that differ from those of the original cells. In contrast, K4DT‐immortalized cells retain their original cellular characteristics (Bai et al. 2023; Bai et al. 2024). These advances in the K4DT method suggest that K4DT‐immortalized cells are more suitable for applications that require genetic stability and long‐term culture.

In the present study, we aimed to establish immortalized bovine granulosa cells by applying the K4DT method. Generation of such bGC‐K4DT cells is anticipated to provide a novel and practical system for evaluating the activation potential of PRP in ovarian follicles. This model will facilitate exploration of the mechanisms of action of PRP, thus contributing to advances in management of cattle reproductivity and overall livestock productivity.

## 2 Materials and Methods

### 2.1 Virus Preparation

Recombinant virus was produced using the packaging plasmids pCAG‐HIVgp and pCMV‐VSVG‐RSV‐Rev, which were graciously provided by Hiroyuki Miyoshi of the RIKEN BioResource Center (Tsukuba, Japan) for lentiviruses, and pCL‐HIVgp (Gag and Pol) and pCMV‐VSVG for retroviruses. 293T cells were co‐transfected with one of the following expression vectors: CSII‐CMV‐CDK4^R24C^, CSII‐CMV‐cyclin D1, CSII‐CMV‐TERT, CSII‐CMV‐EGFP, PQCXIN‐SV40, or PQCXIN‐EGFP, and corresponding packaging plasmids using polyethyleneimine‐max. Two days post‐transfection, the medium was replaced with fresh medium containing 10 μM forskolin. At 72 h post‐transfection, the viral fluids filtered through a 0.45 μM disk filter were mixed with PEG6000 (320 mg/mL) at a ratio of 3:1 (v/v). Viral particles were concentrated by centrifugation at 2190*g* for 60 min at 4°C, and the resulting viral pellets were resuspended in 1.5 mL of fresh medium containing 4 μg/mL polybrene for subsequent experiments.

### 2.2 Cell Preparation and Infection

Granulosa cells were collected from ovaries obtained from cattle at the time of slaughter at the Meat Processing and Storage Center. Medium‐sized follicles (5–8 mm) were dissected, and mural granulosa cells were isolated by opening each follicle and gently scraping the inner (theca‐facing) surface of the follicular wall. To exclude cells derived from immature follicles, non‐adherent (floating) cells were removed after 4 h of culture, and only the cells attached to the culture dish were collected. Two types of bGCs were isolated from the ovaries and cultured in 6‐well plates coated with 10% CellMatrix (Nitta Gelatin Co. Ltd. Osaka, Japan) using DMEM/Ham's F‐12 medium with high glucose (FujiFilm Wako Pure Chemical Co., Osaka, Japan) supplemented with 10% fetal bovine serum (Mediatech, Unc. (DBA J R Scientific), CA, USA; Lot 27419001), 1× antibiotic mixture (Sigma‐Aldrich, St. Louis, MO, USA), and ZellShield (Minerva Biolabs GmbH, Berlin, Germany; Lot 130S1116). Primary bGCs (3 × 10^5^ cells per well) were precultured and subsequently infected with concentrated virus in the presence of polybrene (4 μg/mL). As described previously (Donai et al. 2014; Fukuda et al. 2018; Kuroda et al. 2015), the infected cells were referred to as K4D, K4DT, SV40, and enhanced green fluorescent protein (EGFP) cells, reflecting the genes introduced into them. EGFP‐expressing lentivirus/retrovirus infection was efficiently validated via fluorescence imaging.

### 2.3 PCR and Western Blot Analysis

Genomic DNA was extracted from all cell types using the NucleoSpin Tissue kit (Takara Bio, Shiga, Japan) according to the manufacturer's protocol. Each of the introduced genes, along with the bovine endogenous tuberous sclerosis complex 1 (TSC1) gene, was amplified from genomic DNA using KOD FX Neo polymerase (TOYOBO, Osaka, Japan). The primer sets used are listed in Table 1.

**Table 1:** Primer sets for detection of transgenes.

	Sequence	
Target gene	Forward primer (5′ to 3′)	Reverse primer (5′ to 3′)
CDK4	CTTCCCATCAGCACAGTTCGTGAGG	AAAGATTTTGCCCAACTGGTCGGCTTC
cyclin D1	CCCGATGCCAACCTCCTCAACGAC	ATGATCTGTTTGTTCTCCTCCGCCTCTG
hTERT	CTGCTCCTGCGTTTGGTGGATGATT	GTCCTGAGTGACCCCAGGAGTGGCA
SV40 T large antigen	ATGTATAGTGCCTTGACTAGAGATCCAA	CCAGCCATCCATTCTTCTATGTC
bovine TSCI	CTTCGACTCACCCTTCTACCG	TGAAGGCTTGCTTTGGTGTG

Cell lysates were obtained by sonication from each cell type using a homogenization buffer composed of 50 mM Tris–HCl (pH 7.3), 150 mM NaCl, 1% Triton X‐100, 2.5 mg/mL sodium deoxycholate, and protease inhibitors. Concentrations of proteins in the cell lysates were measured using a bicinchoninic acid (BCA) assay kit (Thermo Fisher Scientific, Waltham, MA, USA). Each cell lysate (10 μg) was loaded onto either an 8% or 10% SDS‐PAGE gel. After electrophoresis, proteins were transferred and detected using specific primary antibodies: anti‐CDK4 (mouse monoclonal, Santa Cruz Biotechnology, sc‐56277, 1:2000), anti‐cyclin D1 (rabbit monoclonal, Medical & Biological Laboratories, code no. 553, 1:5000), anti‐SV40 T antigen (mouse monoclonal, Medical & Biological Laboratories Co. Ltd., code no. 147, 1:2000), and anti‐α‐tubulin (mouse monoclonal, Santa Cruz Biotechnology, sc‐32293, 1:1000) as a loading control. Secondary antibodies included anti‐mouse IgG (Medical & Biological Laboratories Co. Ltd., code no. 330, 1: 2500 dilution) and anti‐rabbit IgG polyclonal (Medical & Biological Laboratories Co. Ltd., code no. 458, 1: 2500 dilution).

### 2.4 Cell Division

Parental and transduced cell types were seeded at 4 × 10⁴ cells per well (seeding number, S), and all the cell types were harvested when any one type reached confluence. The total number of collected cells (C) was counted, and the cells were serially passaged. Cell population doubling (PD) was calculated using the formula: PD = log₂(C/S).

### 2.5 Cell Cycle and Senescence Analysis

At passages 2 and 13, cell cycle phases of parental and transduced cells were assessed as described previously (Bai et al. 2024). Senescence‐Associated β‐Galactosidase (SA‐β‐Gal) activity was evaluated in parental (passages 7 and 8), K4D (passages 17 and 22), K4DT (passages 17 and 22), and SV40 (passages 6 and 8) cells using a commercial senescence detection kit (BioVision, Milpitas, CA) according to the manufacturer's protocol.

### 2.6 Stretch PCR Assay

Telomerase activity was assessed using the TeloChaser kit (TOYOBO, Osaka, Japan) as per the manufacturer's protocol. The telomerase products generated from each cell lysate were amplified and analyzed using 12.5% nondenaturing polyacrylamide gel electrophoresis.

### 2.7 Reverse Transcription and Quantitative RT‐PCR

Parental cells, K4DT cells, and bovine fibroblast cells were stimulated with 10 IU/mL human recombinant FSH (hFSH) (Fuji Pharma CO. Ltd., Tokyo, Japan) or with 50 μM forskolin as a positive control for 48 h, after which total RNA was extracted using the NucleoSpin RNA Plus kit (TaKaRa Bio, Tokyo, Japan) according to the manufacturer's instructions. DNase‐treated total RNA was reverse transcribed into cDNA using the PrimeScript RT Reagent Kit with gDNA Eraser (TaKaRa Bio Inc.) as per the manufacturer's protocol. Subsequently, expression levels of the aromatase enzyme, cholesterol side‐chain cleavage enzyme (P450scc), FSH receptor, luteinizing hormone (LH) receptor and glyceraldehyde‐3‐phosphate dehydrogenase gene (*GAPDH*; used as an internal control) were amplified and quantified using real‐time PCR (ClonTech Inc., Tokyo, Japan). Briefly, cDNA samples were mixed with each detection primer (0.3 μM), probe (0.2 μM), and Thunderbird Probe qPCR Mix (ToYoBo, Osaka, Japan) to prepare a PCR reaction mixture to amplify aromatase. The PCR reaction was carried out employing the following conditions: initial denaturation at 95°C for 10 s, followed by 45 cycles of denaturation at 98°C for 10 s and annealing/extension at 60°C for 45 s. For amplification of the remaining genes, thunderbird SYBR qPCR Mix (ToYoBo, Osaka, Japan) was used instead of thunderbird probe qPCR Mix, and reactions were performed using a three‐step RT‐qPCR protocol. Initial denaturation was carried out at 95°C for 15 s, followed by 45 cycles of denaturation at 98°C for 10 s, annealing at 58°C for 30 s, and extension at 72°C for 45 s. The primer sets and probe sequences used in this study are listed in Table 2.

**Table 2:** Primer sets and probe for the detection of aromatase, P450scc, FSH receptor, LH receptor, and GAPDH genes.

Target gene	Primer and probe	Sequence (5′ to 3′)	GenBank accession number	Product length (bp)
CYP19A1 (aromatase)	Forward	TCGCCATGGTGATGATGAAG	NM_174305	125
	Probe	FAM_TCGGTGCGTTGAGAAGATGCAGAA_BHQ		
	Reverse	CTCGTCTGGATGCAAGGATAAG		
CYP11A1 (P450scc)	Forward	CGAGCAGAGACAGCAGCAG	NM_176644.2	185
	Reverse	GATCTCACTGTAGGGGCGAG		
FSH receptor	Forward	ATCTCTGCCTCCCTCAAGGT	NM_174061.1	132
	Reverse	ATCCCTGCGGAAGTTCTTGG		
LH receptor	Forward	CATGGACATTGACAGCCCCT	NM_174381	165
	Reverse	ATGCGCTTGGCTATCTTGGT		
GAPDH	Forward	AGGAGCACGAGAGGAAGAGT	NM_001034034.2	147
	Reverse	TGATGGTACACAAGGCAGGG		

### 2.8 Karyotype Analysis

The karyotypes of the K4DT cells were analyzed as follows. Cells at approximately 70%–80% confluence were treated overnight with 50 ng/mL Colcemid to arrest them in metaphase. The cells were then collected, treated with 75 mM KCl, and fixed with a mixture of acetic acid and methanol. The fixed cells were placed on glass slides, air‐dried, and stained with Giemsa. Chromosomes were observed under a KEYENCE microscope, and their numbers were counted manually.

### 2.9 Statistical Analysis

Effect of the cell cycle phase was assessed using analysis of variance (ANOVA) followed by Tukey's post hoc multiple comparison test. A *p* value less than 0.05 was considered statistically significant. The levels of significance are denoted as follows: *p < 0.05, **p < 0.01, and ***p < 0.001.

## 3 Results

### 3.1 Recombinant Lentiviral‐Mediated Gene Delivery

**Figure 1:**
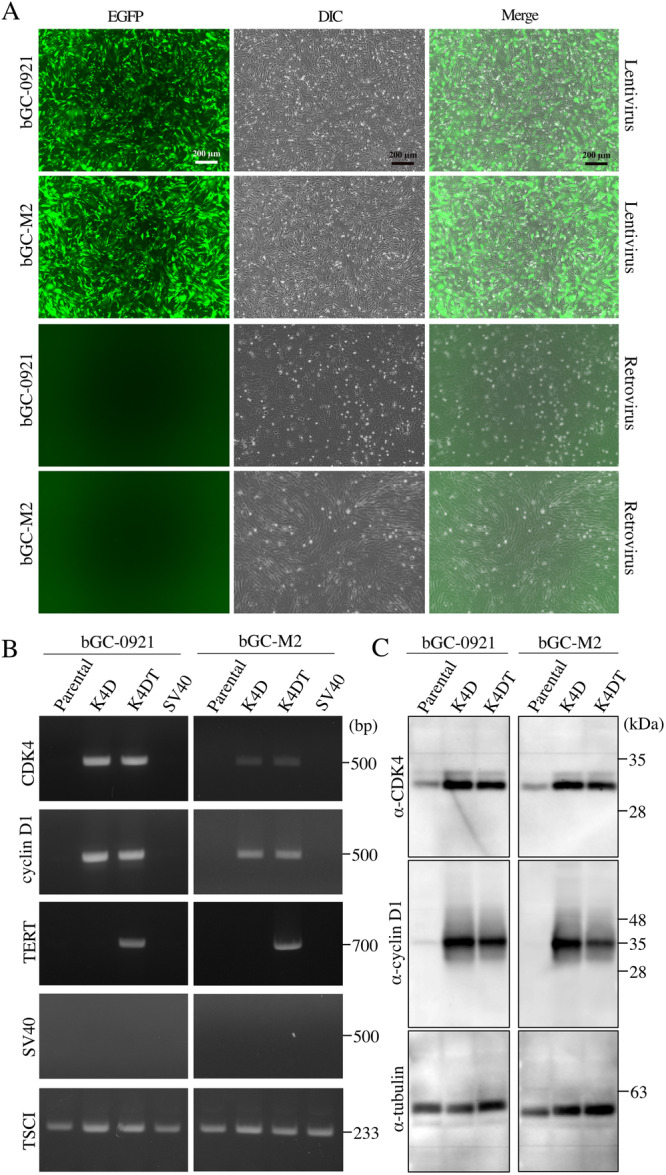
Detection of transgenes in bovine granulosa cells (bGCs). (A) Assessment of EGFP expression to evaluate infection efficiency. Parental bGCs were transduced with EGFP‐expressing retrovirus or retrovirus for 72 h, and expression of EGFP was subsequently visualized. Scale bar: 200 μm. (B) PCR‐based detection of transgenes integrated into genomic DNA. Genomic DNA extracted from parental and transduced bGCs was subjected to PCR amplification targeting the introduced genes. Endogenous TSCI gene served as an internal control. Molecular weights (in base pairs) are indicated on the right side of the agarose gel images. (C) Western blot analysis of transgene‐encoded protein expression. Cell lysates from parental and transduced bGCs were analyzed by western blot analysis using primary antibodies specific to the introduced proteins. Tubulin was used as an internal loading control. Protein molecular weights (in kDa) are shown adjacent to the bands.

Parental bGC cells were transduced with harvested recombinant viruses as described in the Section 2. Efficiency of infection was assessed by measuring the expression of EGFP, which serves as a marker of successful viral transduction. As shown in Figure 1A, both types of bGCs exhibited high infection efficiencies following transduction with the recombinant viruses. This indicates that bGCs were effectively transduced with the lentiviral vectors carrying mutant CDK4, cyclin D1, or TERT. In contrast, no EGFP expression was observed in cells infected with the retrovirus that encoded EGFP in its recombinant construct. This suggests that the SV40 large T antigen produced by the retrovirus failed to mediate successful integration of the transgene into bGCs. The retroviral infection experiment was independently repeated thrice, consistently yielding identical results.

### 3.2 Analysis of Transgenes and Protein Expression

To verify the presence of transgenes, genomic DNA from each cell type was subjected to PCR using gene‐specific primer sets. Amplification products of expected sizes were specifically observed for CDK4 and cyclin D1 in K4D cells, and for CDK4, cyclin D1, and hTERT in K4DT cells (Figure 1B). These results suggest that cell cycle regulator genes were successfully introduced into the transduced bGCs. Bovine TSCI gene served as endogenous control gene. As anticipated, the SV40 T large antigen gene was not amplified from bGCs infected with the retrovirus encoding this gene. These findings further confirm that bGCs are refractory to retrovirus‐mediated delivery or stable integration of the SV40 T large antigen. Furthermore, transgene expression was assessed at the protein level. As depicted in Figure 1C, CDK4 was detected at the expected molecular weight in both parental and their transduced cells (K4D and K4DT, respectively), with significantly higher expression observed in the transduced cells. Similarly, cyclin D1 expression was specifically detected in the transduced cells. Tubulin was used as the loading control. Results of western blot analysis revealed that the transgenes were successfully expressed as proteins.

### 3.3 Cell Cycle Phase Distribution

**Figure 2:**
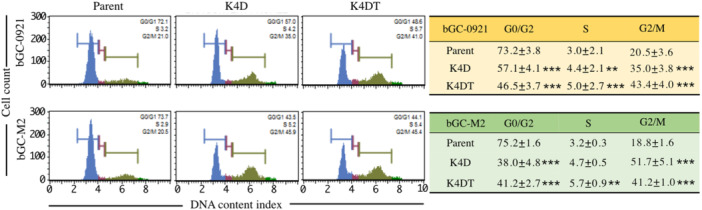
Cell cycle analysis of bGC‐derived cells. Each cell type (1 × 10⁵ cells) was collected, fixed, and stained for cell cycle analysis. Representative histograms illustrate distribution of cells across different phases of the cell cycle. Quantitative results show the percentage of parental and transduced cells in each phase. Data are presented as mean ± standard error (SE), based on triplicate experiments for each sample. Statistical differences between groups were assessed using Tukey's multiple comparison test, with significance denoted in the bar graphs by asterisks (**p < 0.01, ***p < 0.001).

Two transgenes that encode CDK4 and cyclin D1, play essential roles in regulating the transition from the G1 to S phase of the cell cycle. Accordingly, cell cycle phase distribution was analyzed in both parental and transduced cells. The proportions of cells in the G0/G1, S, and G2/M phases for each cell type are presented in Figure 2. Each type of the transduced cells exhibited more rapid progression through the S phase than the parental cells, bGC‐0921 and bGC‐M2. Additionally, the proportion of cells in the G2/M phase was significantly higher in all the transduced cell types than in parental cells, indicating enhanced advancement of the cell cycle. Significant differences in the G1/G and G2/M phase distributions were observed between the transduced bGC‐0921‐K4D and bGC‐0921‐K4DT cells but not in the transduced cells derived from bGC‐M2. These findings suggested that the G1 to S phase transition was promoted in all the transduced cell types, resulting in accumulation of cells in the G2/M phase.

### 3.4 Assessment of Cell Proliferation and Senescence

**Figure 3:**
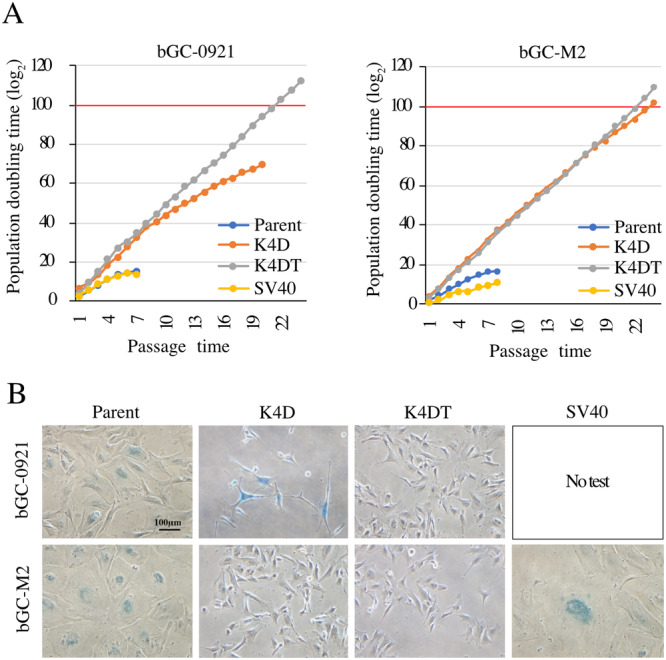
Assessment of cell proliferation and senescence in bGC‐derived cells. (A) Population doubling levels were evaluated for parental and transduced bGC‐derived cells. Each cell type was seeded in 6‐well plates and cultured until one type reached full confluence. The cells were then harvested and counted. Population doubling levels were calculated using the formula: log₂ (final cell number/initial seeding number). (B) Cellular senescence was assessed by senescence‐associated β‐galactosidase (SA‐β‐gal) staining. Positive senescent cells were stained blue. Parental, K4D‐0921, and M2‐SV40 cells exhibited blue color, indicating senescence, whereas K4DT and M2‐K4D cells were negative for SA‐β‐gal staining, suggesting a non‐senescent state. Scale bar:100 μm.

Primary cells have a limited proliferative lifespan, presenting a significant challenge for extended studies. To address this issue, proliferative capacity of all the cell types was evaluated by calculating the PD time. Kyo et al. have identified PD 100 as a threshold point for defining cellular immortalization (Kyo et al. 2003). As shown in Figure 3A, the parental cells, bGC‐0921 and bGC‐M2, achieved a PD time of PD15 and PD16, respectively. Conversely, the three transduced cell types (bGC‐0921‐K4DT, bGC‐M2‐K4D, and bGC‐M2‐K4DT) surpassed PD100, indicating that these cells were successfully immortalized. Notably, bGC‐0921‐K4D cells proliferated only up to PD68. The only difference between bGC‐0921‐K4D and bGC‐0921‐K4DT was the presence of hTERT. The failure of bGC‐0921‐K4D to achieve immortalization suggests that telomere shortening may have limited their proliferative capacity.

Cytoplasmic enlargement was evident in both the parental and transduced bGC‐0921‐K4D cells, representing a hallmark of the senescent phenotype (Figure 3B). In addition, all the cell types were subjected to SA‐β‐Gal staining, a well‐established biomarker for senescence. SA‐β‐Gal activity corresponds to elevated lysosomal β‐galactosidase levels that accumulate in senescent cells and produce a characteristic blue stain, distinguishing them from proliferating or quiescent cells. Blue staining was observed in both the parental and transduced bGC‐0921‐K4D cells, but not in the other three transduced cell types (PD > 100), suggesting that the latter three types of transduced cell were immortalized without any signs of cellular senescence.

### 3.5 Telomerase Activity

**Figure 4:**
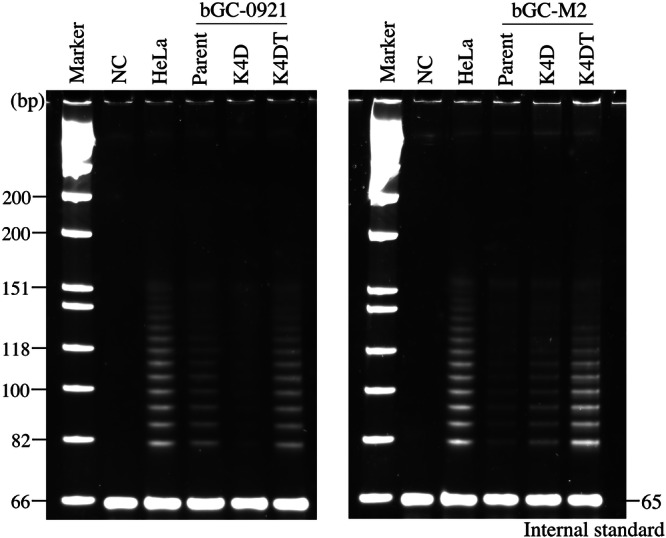
Assessment of telomerase activity in bGC‐derived cells. Cell lysates from parental and transduced bGC‐derived cells were analyzed for telomerase activity using a stretch PCR‐based assay. HeLa cells were included as a positive control for telomerase activity.

Telomere shortening represents a major limitation to prolonged proliferation and expansion of mammalian cells in culture (Deng et al. 2008). Among the transduced cell types, bGC‐0921‐K4D cells failed to achieve immortalization, whereas bGC‐0921‐K4DT cells successfully achieved this capacity. To clarify the underlying cause, telomerase activity was assessed in the parental and all transduced cell types. As shown in Figure 4, both the early‐passage parental bGC‐0921 (p2) and bGC‐0921‐K4DT cells (p19) exhibited robust telomerase activity. In contrast, bGC‐0921‐K4D cells (p19) displayed weak telomerase activity, suggesting that insufficient telomerase activity may have contributed to their failure to be immortalized. Parental bGC‐M2 cells (p4) exhibited weaker telomerase activity than their transduced counterparts. Notably, although bGC‐M2‐K4D cells (p19) displayed telomerase activity, the bGC‐M2‐K4DT cells (19) demonstrated the highest enzymatic activity, which was comparable to that of the positive control, HeLa cells. The effectiveness of telomerase activity was as follows: parental cells < bGC‐M2‐K4D < bGC‐M2‐K4DT. These findings support the positive correlation between telomerase activity and proliferative capacity of the cells.

### 3.6 Gene Expression in bGC‐Derived Cells

**Figure 5:**
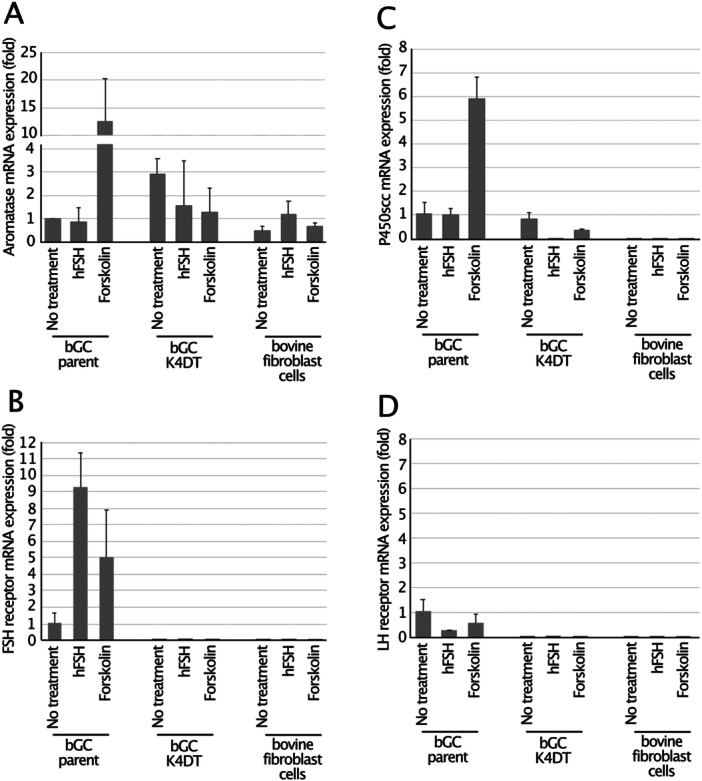
Comparison of gene expression at mRNA level among parental bGCs, transduced K4DT, and bovine fibroblast cells. (A) Relative expression of aromatase (CYP19A1); (B) relative expression of FSH receptor; (C) relative expression of P450scc (CYP11A1); (D) relative expression of LH receptor. Total RNA was extracted from parental bGCs, transduced K4DT, and bovine fibroblast cells following stimulation with either forskolin or human recombinant FSH (hFSH), and then reverse transcribed into cDNA. Aromatase gene expression was quantified using qRT‐PCR. The expression levels were normalized to GAPDH as an endogenous control, and calculated using the comparative CT (∆∆CT) method. The expression level in the parental bGCs under untreated condition was set as 1 for comparison. The vertical axis represents relative expression level of the aromatase gene, while the horizontal axis indicates the different cell types. Error bars represent standard deviation from three independent experiments.

Steroidogenic enzymes and gonadotropin receptors exhibit characteristic expression patterns that together define granulosa‐cell identity, steroidogenic capacity, and follicular maturation. Aromatase drives estradiol synthesis by converting androgens, and P450scc initiates steroidogenesis through cholesterol conversion. In parallel, the FSH receptor mediates FSH‐dependent proliferation and estrogen production, while LH receptor expression is acquired during the advanced stages of follicular development. To assess aromatase expression in bGCs and K4DT cells, primers and a probe were designed based on the gene encoding the cytochrome P450 family 19 subfamily A member 1 (CYP19A1; Accession No. NM_174305). Total RNAs was extracted from cells that had been cultured via stimulation with forskolin and hFSH. Forskolin is commonly used in vitro to mimic FSH signaling and robustly induces aromatase expression through the cAMP/PKA pathway. Aromatase gene expression was measured using quantitative real‐time PCR (qRT‐PCR). Aromatase mRNA levels were normalized to GAPDH mRNA level, which served as an internal control, and analyzed using the comparative CT (∆∆CT) method. The expression level of parental bGCs under untreated condition was used as a reference and set to 1 for comparison. Parental bGCs showed high aromatase when treated with forskolin. In contrast, both the parental bGCs and K4DT cells exhibited low levels of aromatase expression upon stimulation with hFSH. Notably, aromatase expression was also observed under untreated conditions and these levels remained largely unchanged after hFSH treatment (Figure 5A). In contrast, bovine fibroblast cells displayed consistently lower aromatase expression regardless of treatment, further supporting the notion that aromatase expression is specific to parental bGCs and their derived K4DT cells. In K4DT cells, aromatase expression levels were comparable between the two treatments. These results indicate that bGC‐derived immortalized K4DT cells retain aromatase expression, suggesting that they maintain a steroidogenic phenotype and have the potential for estrogenic activity. Further, parental bGCs exhibited high FSH receptor expression when stimulated with either hFSH or forskolin, whereas bGC‐K4DT cells showed no detectable FSH receptor expression (Figure 5B). These findings suggest that cAMP elevation can indirectly stabilize or maintain FSH receptor transcription. P450scc, encoded by the cytochrome P450 family 11 subfamily A member 1 gene (CYP11A1), was strongly induced by forskolin in parental bGCs but not in the other cell types examined (Figure 5C). In contrast, LH receptor expression remained low in parental bGCs (Figure 5D), indicating that LH receptor transcription requires additional differentiation signals that are not reproduced by forskolin stimulation alone. The data indicated that parental bGCs maintain key granulosa‐cell characteristics and a functional cAMP–responsive steroidogenic program, whereas bGC‐K4DT cells have lost these granulosa‐cell features.

### 3.7 Karyotype Distribution of the Immortalized bGC Cells

**Figure 6:**
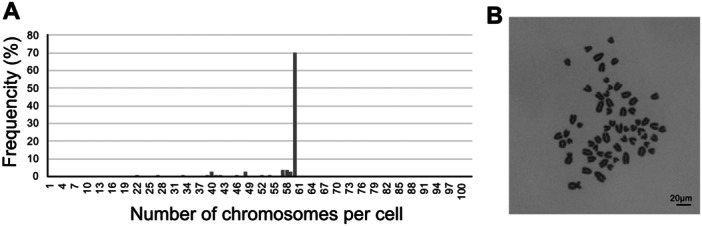
Karyotypes analysis of K4DT cells. (A) Chromosome number distribution. Metaphase‐arrested K4DT cells were fixed and Giemsa‐stained. Karyotypes were imaged and manually analyzed (*n* = 102). Chromosome number frequencies are shown as histograms. (B) Representative chromosomal pattern. Scale bar: 20 μm.

The genetic stability of the established cells is a critical factor in ensuring their reliability. A normal chromosomal pattern indicates that the cells have retained their original genetic integrity. Therefore, the karyotype distribution of the established bGC‐K4DT cells was analyzed. The chromosome number of the parental bGCs was not examined because the cells underwent senescence and exhibited telomere shortening, making it difficult to obtain a sufficient number of cells for karyotype analysis. In contrast, the K4DT cells were successfully analyzed. Cattle possess a diploid chromosome number of 2n = 60, and approximately 70% of bGC‐K4DT cells (*n* = 102) exhibited a normal chromosomal pattern, as shown in Figure 6A. A representative karyotype image is presented in Figure 6B. These findings indicate that the established bGC‐K4DT cells maintained their original stable karyotype distribution as the parental cells.

## 4 Discussion

Cell immortalization is characterized by the circumvention of normal proliferative limits, enabling continuous cell division. This is typically achieved by inserting viral genes or activating oncogenes, which allow cells to bypass the usual cell cycle checkpoints. Previous studies have shown that luteinized granulosa cells cannot be fully immortalized by introducing mutant CDK4^R24C^ and cyclin D1, along with TERT alone (Yang et al. 2017). However, inclusion of additional oncogenes, such as HPV16 E6E7, E6, or mutant p53^C234^, has been found to enable their immortalization (Hang et al. 2016; Opitz et al. 2001). Furthermore, transduction with SV40 large T antigen has been shown to immortalize yak granulosa cells (Wen et al. 2024). Some reports also suggested that combined expression of cell cycle regulators, CDK4^R24C^ and cyclin D1, with TERT successfully immortalized cells from various mammalian species (Bai et al. 2023; Bai et al. 2024; Fukuda et al. 2021; Kikuchi et al. 2023; Furuya et al. 2021; Orimoto et al. 2020; Gouko et al. 2018). These evidences suggest that either the combined expression of CDK4^R24C^, cyclin D1 with TERT, or the SV40 large T antigen, could be used to establish immortalized bGCs. In the present study, all of these factors except the SV40 large T antigen were transduced into primary bGCs to achieve immortalization.

Two types of bGCs were successfully infected using lentiviral vectors encoding mutant CDK4, cyclin D1, and TERT, whereas retroviral vectors expressing SV40 large T antigen failed to infect the bCGs. Both the viral systems employed the same packaging plasmid, pCMV‐VSVG‐RSV‐Rev, to generate the envelope protein. Given that the lentivirus was capable of infecting bGCs, it can be inferred that these cells possess appropriate receptors for VSVG‐mediated viral entry. Consequently, we anticipated that a retroviral vector bearing the SV40 large T antigen would also be capable of infecting bGCs under the same conditions. Hence, a retrovirus encoding the SV40 large T antigen was prepared and used employing the same protocol as that for infecting other target cells, and it resulted in a high infection rate (Bai et al. 2023, 2024). This indicated that, in this context as well, the titers of the retrovirus were sufficient to infect the bCGs. This result implies that the failure of retroviral transduction in the present study was not due to an insufficient viral titer. Thus, failure of retroviral transduction suggests limitations beyond receptor binding. PCR amplification of the SV40 large T antigen gene was unsuccessful in both types of bGCs following infection with retroviral vectors encoding the antigen. Additionally, EGFP expression was not detected in bGCs infected with retroviral vectors carrying the EGFP gene. Lentiviruses differ from other retroviruses primarily in their ability to integrate efficiently and randomly into the genome of host cells, including non‐dividing cells (Trobridge and Russell 2004). In contrast, conventional retroviruses require host cell mitosis for successful integration, as they depend on breakdown of the nuclear envelope and tend to integrate near transcription start sites (Kvaratskhelia et al. 2014). Therefore, failure of retroviral transduction may have resulted from unexpected events occurring during the M phase in parental bGC cells. These findings suggest that although the retroviral particles successfully delivered their cargo to the bGCs, the transgenes—SV40 large T antigen and EGFP—failed to integrate into the host genome.

PD values of both the SV40‐transduced cell types were lower than those of their parental bGC cells, likely due to cytotoxicity and stress associated with infection using a high concentration of retrovirus. Notably, cellular senescence and damage progressed more rapidly in bGC‐0921‐SV40 cells compared to their parental counterparts, which resulted in an insufficient number of cells to perform SA‐β‐Gal staining. A comparable effect was also noted in the bGC‐M2‐SV40 cells; however, SA‐β‐Gal test was performed using a small number of cells. Typical features of cellular senescence, such as enlarged cytoplasm, were observed in both parental and SV40‐transduced cells. Notably, the SV40‐transduced cells exhibited more pronounced cytoplasmic enlargement, suggesting a more advanced state of senescence.

Typically, most of primary mammalian cells undergo approximately 40–60 divisions during their lifespan. However, granulosa cells are specialized follicular cells; therefore, their proliferative capacity is limited compared to other cell types. In this study, the two parental bGC cells divided only 14 and 16 times, respectively. Therefore, these parental bGC cells are believed to undergo roughly a dozen divisions to proliferate until the end of their lifespan, ultimately entering senescence. Consequently, the transduced cells achieved a PD value of 100, which is commonly considered indicative of successful immortalization (Kyo et al. 2003). Three types of bGC‐derived cells—bGC‐0921‐K4DT, bGC‐M2‐K4D, and bGC‐M2‐K4DT—were fully immortalized. Notably, bGC‐M2‐K4D cells exhibited a PD value exceeding 100, indicating sustained proliferative capacity, which was not observed in bGC‐0921‐K4D cells. Despite their high proliferation rate, bGC‐M2‐K4D cells showed lower telomerase activity than bGC‐M2‐K4DT cells. These findings suggest that the parental bGC‐M2 cells possessed stronger telomerase activity. Furthermore, telomerase activity was measured using early‐passage parental bGC‐0921 cells, as the late‐passage cells had stopped dividing and could not yield sufficient material for analysis. Results suggested that the early‐stage cells demonstrated strong telomerase activity. Additionally, the established bGC‐K4DT cells displayed a normal karyotype distribution, indicating genomic stability, maintenance of original cellular properties, and suitability for both research and industrial applications.

Parental bGCs express aromatase, a key enzyme involved in ovarian function, and this activity is also preserved in immortalized bGC‐K4DT cells. Upon stimulation with or without hFSH, aromatase expression in parental bGCs was approximately 12‐fold lower than that in forskolin‐treated cells. Notably, bGC‐K4DT cells exhibited comparable levels of aromatase expression when treated with either forskolin or hFSH relative to parental bGCs treated with hFSH. In contrast, a study reported that hFSH robustly activated aromatase expression in human granulosa cells (Bayasula et al. 2012). These findings suggest that the use of bovine recombinant FSH instead of hFSH may enhance aromatase expression in bGCs. An attempt was made to obtain bovine recombinant FSH from overseas sources; however, unfortunately, it was not available. Aromatase activity is strongly associated with follicular development, especially during selection and growth of the dominant follicle (Hillier 2001). Small antral follicles display minimal or undetectable levels of aromatase mRNA and protein (Macklon and Fauser 2001). At this early developmental stage, granulosa cells remain insufficiently stimulated by FSH, which is essential to trigger aromatase expression (Stocco 2008). Therefore, it is assumed that the parental bGCs were derived from early‐stage or small antral follicles. It is well established that aromatase expression in granulosa cells declines sharply under in vitro culture conditions and often becomes undetectable without continuous hormonal support or follicular microenvironmental cues (Picton et al. 1999). Consistent with these reports, both the parental bGCs and K4DT cells in our study showed very low to undetectable aromatase expression, even after stimulation with forskolin or hFSH. However, the lack of detectable FSHR and LHR expression indicates that the immortalization process likely resulted in transcriptional silencing or epigenetic repression of these gonadotropin receptor genes. Given that FSHR expression is typically upregulated during granulosa cell differentiation and follicular development, its absence is indicative of a dedifferentiated or progenitor‐like cellular state. Nina et al. also reported that immortalized GCs exhibit aberrant gonadotropin receptor expression, thereby complicating the physiological interpretation of experimental findings (Alyoshina et al. 2025). Moreover, as CYP11A1 encodes the enzyme responsible for the initial and rate‐limiting step of steroid hormone biosynthesis, the absence of its expression suggests that the bGC‐K4DT cells exhibit a non‐steroidogenic phenotype. These suggest that immortalization preserved some granulosa‐like features but not the full steroidogenic capacity observed in vivo. As models for investigating follicular development, these known regulatory factors can be introduced as transgenes via vector‐mediated gene transfer into the immortalized bGC‐K4DT cells. Genetic manipulation of these immortalized cells allows controlled expression of key folliculogenic regulators, thereby enabling the dissection of their individual and combined roles in granulosa cell function. Direct measurement of estrogen (E2) levels would offer a more precise assessment of granulosa cell function than evaluating aromatase gene expression alone. However, due to the unavailability of LC‐MS/MS or other hormone measurement instruments, estrogen analysis was not conducted in this study. To address this limitation, we plan to measure estradiol levels in conditioned media in future work—initially using a validated ELISA and, where possible, LC‐MS/MS through collaborative access—to directly confirm the steroidogenic function of the immortalized granulosa cells.

PRP contains various growth factors such as PDGF, TGF‐β, VEGF, and EGF, which are known to promote cell proliferation, reduce apoptosis, and enhance tissue regeneration (Lange‐Consiglio et al. 2023). Granulosa cells express receptors for several of these factors, and their activation can stimulate cell proliferation and hormone production (Samira et al. 2019). Previous studies have demonstrated that PRP enhances granulosa cell activity and supports follicular development both in vitro and in vivo. Therefore, PRP may act on granulosa cells by activating common cell‐survival and growth pathways, thereby improving the follicular microenvironment and promoting oocyte maturation (Samira et al. 2019). Additionally, the development of comprehensive in vitro models that closely mimic the ovarian microenvironment should also be a focus of study. It would allow researchers to systematically investigate the specific bioactive components of PRP responsible for follicular activation and growth. Moreover, exploring the interactions between PRP and other reproductive hormones or signaling pathways could provide deeper insights into its mechanism of action. By addressing these knowledge gaps, it will be possible to refine PRP‐based protocols and establish evidence‐based guidelines for its application in cattle reproduction.

Although several granulosa cell lines have been developed to study follicular function and steroidogenesis, most originate from rodent or porcine models and often exhibit limited functional fidelity or genomic instability. For instance, the porcine PGV line (SV40T‐immortalized) survived long‐term culture but lacked progesterone synthesis and P450scc expression (Lin 2005), while the bovine BGC‐1 line showed altered growth‐factor responses and minimal steroidogenic activity (Lerner et al. 1995). A yak granulosa line (yGC‐SV40T) retained marker expression and hormone synthesis but remains species‐distant from cattle (Wen et al. 2024). In contrast, our newly established bGC‐K4DT cells offer key advantages: it maintains the normal diploid karyotype (2n = 60) with high genomic stability, preserves a vital enzyme aromatase, and supports active ovarian function. This species‐relevant, functionally stable, and genetically consistent line provides a robust platform for bovine granulosa cell research, follicular physiology, and potential biotechnological applications.

## 5 Conclusion

This study describes development of one K4D‐immortalized and two K4DT‐immoralized bGC cells, achieved through simultaneous expression of CDK4^R24C^ and cyclin D1 along with TERT. In contrast, a widely used immortalized method—SV40 T large antigen transduction—failed to introduce transgenes into parental bGC cells. All the three types of immortalized cells exhibited extended lifespans without signs of cellular senescence, and maintained cell cycle profiles similar to those of the parental cells. The immortalized bGC‐K4DT cells retain aromatase expression, albeit at reduced levels, thereby providing a reliable and practical in vitro model for investigating estrogen biosynthesis. In contrast, the absence of FSH receptor, LH receptor, and P450scc expression indicates a lack of functional steroidogenic capacity. Furthermore, the K4DT method enables the generation of cells from species and tissues that are typically challenging to immortalize using SV40‐based approaches, thereby broadening its applicability in livestock research. By leveraging this technology, researchers can develop robust cellular models to study cattle reproductive biology, optimize PRP applications, and drive innovations in livestock productivity in the future.

## Author Contributions

Lanlan Bai, Tohru Kiyono, and Tomokazu Fukuda conceived and designed the study and wrote the manuscript. Lanlan Bai, Minami Takahashi, Jin Kobayashi, Takahiro Eitsuka, Himari Matsusaka, Taku Ozaki, Eriko Sugano, Hiroshi Tomita, Yuan Xu, Kazuhiro Kawamura, Kiyotaka Nakagawa, Tohru Kiyono, and Tomokazu Fukuda contributed to the data validation and analysis. All the authors have read and approved the final version of the manuscript.

## Funding

The authors received no specific funding for this work.

## Ethics Statement

Primary cells were obtained in full accordance with applicable ethical and legal regulations. All cell cultures used in this study were maintained under standardized laboratory conditions.

## Conflicts of Interest

The authors declare no conflicts of interest.

## Data Availability

All data generated or analyzed throughout the course of this study are included in this article.
